# Dexterity and technique in termite fishing by chimpanzees (*Pan troglodytes troglodytes*) in the Goualougo Triangle, Republic of Congo

**DOI:** 10.1002/ajp.23215

**Published:** 2020-11-16

**Authors:** Antonio J. Osuna‐Mascaró, Camila Ortiz, Caroline Stolz, Stephanie Musgrave, Crickette M. Sanz, David B. Morgan, Dorothy M. Fragaszy

**Affiliations:** ^1^ Messerli Research Institute University of Veterinary Medicine Vienna Wien Austria; ^2^ Department of Psychology University of Georgia Athens Georgia USA; ^3^ Department of Anthropology University of Miami Coral Gables Florida USA; ^4^ Department of Anthropology Washington University in St. Louis Saint Louis Missouri USA; ^5^ Congo Program, Wildlife Conservation Society Brazzaville Republic of Congo; ^6^ Lester E. Fisher Center for the Study and Conservation of Apes, Lincoln Park Zoo Chicago Illinois USA

**Keywords:** handedness, hand postures, manual dexterity, motor skill, tooling

## Abstract

Although the phenomenon of termite fishing by chimpanzees (*Pan troglodytes*) has historical and theoretical importance for primatology, we still have a limited understanding of how chimpanzees accomplish this activity, and in particular, about details of skilled actions and the nature of individual variation in fishing techniques. We examined movements, hand positions, grips, and other details from remote video footage of seven adult and subadult female chimpanzees using plant probes to extract *Macrotermes muelleri* termites from epigeal nests. Six chimpanzees used exclusively one hand (left or right) to grip the probe during termite fishing. All chimpanzees used the same repertoire of actions to insert, adjust, and withdraw the probe but differed in the frequency of use of particular actions. Chimpanzees have been described as eating termites in two ways—directly from the probe or by sweeping them from the probe with one hand. We describe a third technique: sliding the probe between the digits of one stationary hand as the probe is extracted from the nest. The sliding technique requires complementary bimanual coordination (extracting with one hand and grasping lightly with the other, at the same time). We highlight the importance of actions with two hands—one gripping, one assisting—in termite fishing and discuss how probing techniques are correlated with performance. Additional research on digital function and on environmental, organismic, and task constraints will further reveal manual dexterity in termite fishing.

## Research Highlights

‐ Using remote video footage from camera traps in Goualougo Triangle, Republic of Congo, we describe chimpanzees' manual actions, postures, and positions, and movements of the probe while they fished for termites in epigeal termite nests.

‐ Chimpanzees used diverse grips, with and without the thumb, and two hands—one gripping, one assisting—to handle the probe delicately and to move it precisely.

‐ We describe a new technique for recovering termites: sliding the probe between the digits of one stationary hand as the probe is extracted from the nest with the other hand, and a new action: oscillatory movements of the probe while it was inserted in the nest.

## Graphical abstract

chimpanzee fishing termites using the sliding technique.

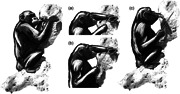

## 1 INTRODUCTION

Termite fishing in chimpanzees (first described by Goodall, 1964) is one of the most widely known examples of tooling (*sensu* Fragaszy & Mangalam, 2018) by a nonhuman animal. Termite fishing is defined as the insertion of a probe (grass, twig, bark, stem, vine, etc.) into a termite nest and withdrawal of the probe with soldiers attached to it by their pinching mandibles. The termites are then consumed by the chimpanzee (Nishida et al., 1999). From eastern to western Africa, wild chimpanzees habitually fish for termites from epigeal (above ground) nests (Mcgrew et al., 1979; Sanz et al., 2004). This behavior is theoretically important to primatologists and other behavioral scientists as traditions vary across chimpanzee populations (Musgrave et al., 2016; Whiten et al., 2001), and because of the potential contributions to chimpanzees' diet conferred by access to this resource (e.g., Deblauwe & Janssens, 2008; O'Malley & Power, 2012). To date, the actions chimpanzees use when fishing for termites have been described in very general terms, and little is known about the dexterity expressed in this activity. In this study, we describe the repertoire of manual actions used in termite fishing by adult and subadult female chimpanzees in the Goualougo Triangle (Sanz & Morgan, 2007; Sanz et al., 2009). The work contributes to the eventual goal of understanding the dimensions and degrees of chimpanzees' dexterity in this activity. Termite fishing is a good candidate behavior for examining details of manual dexterity, as such actions are well‐practiced in adult individuals and one can measure the outcomes of the actions in a variety of dimensions (e.g., rates of feeding; failed attempts; etc.) (e.g., Bardo et al., 2017; Mangalam & Fragaszy, 2015).

Previous reports of hand movements and grips in wild apes have examined actions used during food‐processing, where the handled object is modified for ingestion (e.g., Marzke, 1971; Marzke et al., 2015). In termite fishing, the gripped object is not explicitly modified when in use as a probe, but it is repaired or discarded if it becomes damaged. The goal is to use the object as it is; the task requires delicate movements, not forceful grips. The chimpanzee must control the spatial relation between the probe and the termite nest, rather than its own body. Thus, the manual skills evident during termite fishing differ in character from those evident during feeding on large fruits and meat, as described, for example, by Marzke et al. (2015).

Chimpanzees learn to fish for termites with others in a socially aided setting (i.e., it is a tradition; Lonsdorf, 2005; Musgrave et al., 2016; Whiten et al., 2001). Although there have been experimental analyses of chimpanzees' fishing in captivity using simulations of termite nests (e.g. Hopkins et al., 2009), termite nests visited by chimpanzees in natural habitats are typically larger, more complex structurally, and more variable in many dimensions (e.g., shape, size, and friability of the nest, as well as termite species, density, accessibility, and response) than the simulations achieved in captive studies. The plant materials used as probes likewise vary widely across locations (e.g., exclusive use of bark by chimpanzees in the Issa Valley; Almeida‐Warren et al., 2017), and even between communities at single locations (e.g., Pascual‐Garrido, 2019). It is essential to study this tradition in chimpanzees living in natural conditions in diverse populations to understand the nature of chimpanzees' accommodation to the challenges that termite fishing presents.

### 1.1 Describing manual actions

We generally describe humans' practiced manual actions with objects as skilled or dexterous (Wilson, 1999). Following Bernstein (1967), we hereafter refer to skilled actions in goal‐directed activity in this study as “dexterous.” Dexterity is the ability of an organism to make goal‐directed corrections in movements to optimize effort (Newell, 1986). Performing an action in a more dexterous way allows the individual to reach a goal with a lower expenditure of energy, in less time and with better results than when performing the same action in a less dexterous way (Bernstein, 1967). Dexterity develops with practice as the actor becomes sensitive to the outcome of the preceding movement, or to its modulation (Newell, 1986). Primates' practiced manual action with objects can be examined in terms of dexterity.

Descriptions of dexterous actions with objects in humans include the position of the digits while grasping (grips), in‐hand movements, individuated control of the digits, multidigit synergies, inter‐limb coordination, positional, and postural features, anticipatory movements of the digits during reaching, and kinematics of arm and hand movements (Biryukova et al., 2015; Elliott & Connolly, 1984; Jones & Lederman, 2006; Nonaka & Bril, 2012; Wilson, 1999). For the most complete understanding of dexterity in other primates, descriptions of dexterous actions should use the same variables and concepts that are used to describe dexterous movements in humans. However, at present, our knowledge of several features of dexterous action in nonhuman primates is very limited. Instead, following Napier's (1956, 1960, 1961) seminal comparative works, most of the literature concerning prehension in nonhuman primates has focused on the variety of grips that different species achieve, with particular attention to the role of the thumb (e.g., Christel, 1993; Spinozzi et al., 2004; see Fragaszy & Crast, 2016 for review). The study of manual dexterity in nonhuman primates requires looking beyond static grips to hand movements and the other features of manual action mentioned above.

### 1.2 Organismic constraints on manual action

The morphology of the hands constrains what individuals can do with them. Although there are species in other orders that share the characteristics of primates' hands (unwebbed and long digits with abundant sensitive sensory receptors in the terminal phalanges; Lemelin & Grafton, 1998), only this order is characterized by all of them (Fragaszy & Crast, 2016). The general morphology of the hand and the tactile pads on the palm and the palmar surface of the digits allow primates to use their hands in the exploratory, grasping, and postural ways typical of primates (Fragaszy & Crast, 2016; Jones, 1920). Chimpanzees' hands, as are the hands of other primates, are adapted to climbing (Preuschoft, 1965, 1973), but in this genus are also adapted to terrestrial knuckle walking (Almécija et al., Smaers, & Jungers, 2015; Tuttle, 1969). Their manipulative abilities are constrained by this dual set of adaptations (Lazenby et al., 2011). For example, the morphology of the carpals and metacarpals constrains their ability to cup the palm (Marzke, 1983; Wilson, 1999). Compared with humans, chimpanzees' thenar and hypothenar eminences are smaller and flatter, forearm flexors and interosseous muscles are larger, and thenar muscles are smaller (Ogihara et al. 2005). Chimpanzees' phalanges lack the tufted ends characteristic of humans (Young, 2003), and their fingers are very long in relation to the thumb (Napier, 1960). The phalanges of digits 2–5 in chimpanzees are somewhat curved, rather than straight as in humans. Overall, chimpanzees' hands support suspensory (hook) grips, delicate precision grips, and strong palmar grips with three or more digits wrapped around an object (Marzke & Wullstein, 1996; Napier, 1960).

Haptic perception is critical for dexterous manual action. Haptic sensitivity can be estimated by the density of Meissner's corpuscles, tactile mechanoreceptors in the glabrous skin of primates (Hoffmann et al., 2004). In comparison with human hands, chimpanzees have a lower density of Meissner's corpuscles (Verendeev et al., 2015), a condition that, according to our current understanding, should be interpreted as limiting their manipulative abilities (Hoffmann et al., 2004). As in humans, the density and size of Meissner's corpuscles is similar among chimpanzees' digits, and between the hands (with no relation to hand preference) (Verendeev et al., 2015). These mechanoreceptors are larger (cross‐sectional diameter) in chimpanzees than in marmosets (*Callithrix jacchus*), baboons (*Papio anubis*), and rhesus (*Macaca mulatta*), but most of this size difference is accounted for by the variance in body mass, so the density of chimpanzees' Meissner's corpuscles is similar to that of the other species studied by Verendeev et al. (2015).

As chimpanzees differ from humans in many features of anatomy and sensorimotor organization, they handle objects in a slightly different way than do humans. For example, Crast et al., (2009) note that chimpanzees' repertoire of in‐hand movements (movements that move an object held within the digits, unsupported by the palm; Elliott & Connolly, 1984) is smaller than that of humans, and they perform these movements in a different form than do humans. Nevertheless, they handle objects relatively dexterously, particularly during foraging (Heldstab et al., 2016; Marzke et al., 2015). For example, they manipulate leaves using a broad diversity of actions during food preparation (Stokes & Byrne, 2001), and, when food items have mechanical defenses, they use varied techniques to surmount them (Corp & Byrne, 2002). They use compound grips (different grips in the same hand to hold two or more objects at the same time; Macfarlane & Graziano, 2009) and modify the position of loose objects in one hand with fine digit movements (Boesch & Boesch, 1993; Crast et al., 2009). Like all primates, chimpanzees coordinate movements of the two arms and hands in bimanual actions. They use both symmetric and complementary bimanual actions in foraging and when tooling (Corp & Byrne, 2002; Hopkins, 1995; McGrew, 1974; Sanz & Morgan, 2011).

Chimpanzees use multiple forms of power and precision grips in accord with varying tasks requirements, such as the size and shape of the object and the force and direction of movement they generate with it (Boesch & Boesch, 1993; Marzke & Wullstein, 1996; Marzke et al., 2015; Pouydebat et al., 2011). When picking up a small object such as a raisin from a flat surface, chimpanzees commonly use the lateral side of the index finger to contact the thumb in a precision grip, instead of the pad‐to‐pad contact typical of humans (Christel, 1993; Marzke et al., 1992; Mcgrew et al., 1979; Napier, 1960; Van Lawick‐Goodall, 1971), in accord with their relatively short thumb (Hopkins et al., 2002; Preuschoft, 1973). Chimpanzees in captivity commonly use scissor grips, with the lateral sides of the digits in contact with the object and with the forearm in a supine posture, to grasp and to lift objects 1—2 cm in diameter and about 10 cm in length (Crast et al., 2009 and personal observation). Wild chimpanzees also use this grip (Marzke et al., 2015). To grasp somewhat larger objects, and to move them forcefully, as in striking baobab fruit on a surface, they adapt their grips and motions to the size and weight of the object (Boesch & Boesch, 1993; Marzke & Wullstein, 1996; Marzke et al., 2015; Mcgrew et al., 2005).

Several studies have examined how chimpanzees grip their tools when breaching termite nests, opening access points to beehives, or gathering prey from ant nests (Estienne et al., 2017; Lesnik et al., 2015; Marzke et al., 2015). To perforate the outer crust of an epigeal termite nest (an action requiring forceful pushing) to create an access point to fish for termites, chimpanzees in Goualougo adapt their body position and grips flexibly to the task. They change from power to precision grips depending on the resistance of the crust to penetration by the stick they are wielding, and use their entire body to add force to their actions (Lesnik et al., 2015). Lesnik et al. (2015) describe seven grips used by chimpanzees in Goualougo while perforating epigeal termite nests including interdigital brace, a grip involving three digits. Chimpanzees gathering honey from subterranean bee hives in Loango National Park (Gabon), use different grips and techniques (including feet grips), that vary between individuals (Estienne et al., 2017). When collecting ants, the characteristics of the prey species influence the form and composition of tools and the techniques used by chimpanzees (Humle & Matsuzawa, 2002; Humle, 2010; Mcgrew et al., 2005). Long wands are normally used with two‐handed methods, in which the wand is pulled through one hand with the other hand, whereas when short sticks are used, ants are eaten directly from the tool (Boesch & Boesch, 1990; McGrew, 1974).

### 1.3 Lateral bias in manual actions

Chimpanzees' hand preferences while tooling have been evaluated in several populations. Chimpanzees show a right hand population bias when ant‐dipping, leaf sponging, nut cracking, pestle pounding, algae dipping, and throwing (Biro et al., 2003; Boesch, 1991; Humle & Matsuzawa, 2009; Marchant & McGrew, 2007; Nishida et al., 2013; Sugiyama et al., 1993), but a left hand bias while termite fishing in Gombe (Tanzania) and Fongoli (Senegal) (Bogart et al., 2012; Lonsdorf & Hopkins, 2005; Mcgrew & Marchant, 1992; McGrew & Marchant, 1996). Interestingly, Sanz et al. (2016) report strong hand preferences for each chimpanzee (as have been found in other populations; Bogart et al., 2012; Lonsdorf, 2005; McGrew & Marchant, 1992, 1996, 1999; Nishida & Hiraiwa, 1982; Nishida et al., 1999) in Goualougo, but only a slight right hand bias for this population while fishing termites from epigeal nests. Males had a significant right hand bias, and females showed greater left handedness. This sex difference in the direction of manual bias is consistent with the findings from chimpanzees in Fongoli (Bogart et al., 2012), but not with the chimpanzees at Gombe (Lonsdorf & Hopkins, 2005; Mcgrew & Marchant, 1992). Finally, the Goualougo population showed interesting differences in performance between right‐ and left‐biased chimpanzees. Chimpanzees with a right hand preference, independent of sex, inserted the probe in the nest for shorter periods than chimpanzees with a left hand preference.

### 1.4 Actions used in termite fishing

Chimpanzees in Goualougo fish for termites at both epigeal (above‐ground) and subterranean nests. We restrict our review to behaviors seen when chimpanzees fish at epigeal nests. The individual usually approaches an epigeal nest carrying a stem of an herbaceous plant that will soon be used as a fishing probe. The chimpanzee opens (or most commonly reopens) a termite exit hole on the exterior of the nest with a finger or may pick up a twig to aid in perforating the nest. Once an access point large enough to admit the probe is opened manually or with the perforating tool, if a twig was used for this purpose, the chimpanzee discards the twig, or places it near the feet for reuse later, and grips the herbaceous probe. The probe is usually inserted several inches into the nest. Eventually, termites attack the probe that has invaded the inner chambers of their nest and cling to the probe with their mandibles (Sanz & Morgan, 2007; Sanz et al., 2009). Once extracted, the termites can be eaten directly from the probe or collected from the probe with one hand and eaten from the hand. As part of the tool‐making process, chimpanzees manufacture a brush on one end of the fishing probe, the use of which is associated with increased yield of termites compared to using an unmodified probe (Sanz et al., 2009). They frequently straighten the fibers on the tip of the fishing probe between insertions to facilitate insertion. The chimpanzee inserts and extracts the probe and eats termites clinging to it in repeated quick cycles, with occasional modifications to the tool (Sanz & Morgan, 2011).

The sequence of actions used in fishing for termites (opening a hole, inserting and extracting the probe, and collecting the termites) involves varied hand movements, grips, and degrees of coordination among digits and between limbs (Lesnik et al., 2015). Whereas perforating the hardened surface of an epigeal termite nest can require strong force, sliding the probe in and out of the holes at the surface of the termite nest requires delicate movements of small amplitude and low force. Chimpanzees use complementary and symmetrical bilateral actions when fishing for termites (Humle & Matsuzawa, 2009; Lonsdorf & Hopkins, 2005; Sugiyama et al., 1993). In short, fishing for termites effectively involves long sequences of varied manual actions. Haptic perception and active touch (i.e., perception through touching and handling objects; Lederman & Klatzky, 2009; Turvey, 1996) support these kinds of actions, especially delicate actions with the probe while it is inserted in the nest. Manual dexterity no doubt contributes to effective fishing for termites.

### 1.5 The current study

We approach the question of chimpanzees' manual dexterity while fishing for termites from the perspective of Bernstein (1967), who described dexterity as characterized by efficient, fluid actions in varied circumstances that lead to a desired outcome. This is the usual approach to describing humans' dexterous actions, as described above. We adopt Newell's (Newell & Jordan, 2007; Newell, 1986) expansion of Bernstein's ideas in the constraints‐led perspective. This perspective recognizes that motor skill develops and is expressed in a system encompassing the individual, the setting, and the task. We have previously used this conceptual approach to study how capuchin monkeys handle the challenge of cracking palm nuts using stone hammers, where it is has provided several novel insights (see Fragaszy & Mangalam, 2018 for review). Fishing for termites presents very different constraints (environmental, organismal, and task‐related) than nut‐cracking, and thus serves as an interesting expansion of this approach to the study of tooling. Environmental constraints arise, for example, from the nature of the plant materials available for use as probes, the hardness of the termites' nest, and the characteristics of the termites. Organismal constraints arise from chimpanzee's morphology, size, range of motion of the arms and hands, visuomotor coordination, and so forth. Task constraints arise from the interior structure of the termite nest (e.g., the tunnels' diameter and orientation at the surface, and length of the tunnel to the interior callies where termites gather) and the features of the probes such as length and stiffness. The task requires small movements of low force, there are opportunities to shift grips and move the probe during the action, and the tool must be moved in a delicate way to extract the termites. There are many ways to succeed at this task, and many ways to fail. Using remote video footage, we describe the patterns of chimpanzees' manual actions, limb postures, body positions, and movements of the probe as they fished for termites at epigeal termite nests, to develop a picture of the dexterity that the chimpanzees bring to this task.

## 2 METHODS

### 2.1 Study site

The Goualougo Triangle study area is located in the Congo Basin, northern area of Republic of Congo, along the southern boundary of the Nouabalé‐Ndoki National Park (2°05ʹN–3°03ʹN; 16°51ʹE–16°56ʹE). Its triangular shape is delimited by the Ndoki and Goualougo Rivers, which form the western and eastern boundaries of the area, respectively. The climate in the Goualougo Triangle comprises a transition between the Congo‐equatorial and subequatorial climatic zones (White, 1983) and the area covers 260 km^2^ of lowland forest with altitudes between 330 and 600 m.

All the field protocols, data collection procedures, and data analyses were conducted in accordance with wildlife research protocols, and ethical standards of the Ministry of Science and Technology and the Forest Economy of the Republic of Congo, and the Wildlife Conservation Society of the United States. All research reported in this manuscript complied with the protocols approved by the Animal Care and Use Committee of Washington University in St. Louis, the legal requirements of the Republic of Congo and adhered to the American Society of Primatologists' Principles for the Ethical Treatment of Non‐Human Primates.

### 2.2 Data collection protocols

Sixty‐five remote video monitoring units were used to record chimpanzees fishing for termites at epigeal nests of *Macrotermes muelleri*. Each unit consists of a video camera in a weatherproof case, a passive infrared motion sensor, and custom software designed for the task (CHIMPCAM 1.0 and 2.0; Sanz et al., 2004). The units were located on tree trunks at 0.5–1.5 m above ground level and at 1–5 m from a termite nest. The CHIMPCAM software allows the cameras to record automatically 2 min of video each time that the infrared sensor is triggered, from standby or during a current recording. A selection of videos from 2008 to 2011 were examined.

The videos were screened for light and viewing conditions. Seventeen video clips (30 fps), each around 2 min in duration, of seven individually identified females fishing for termites on epigeal nests were selected for coding. The seven females included five adults (estimated to be more than 13 years old; Maya, Moja, Theresa, Sarah, and Catherine) and two subadults (estimated to be 10–13 years old; Samantha and Dinah). Age‐classes of wild chimpanzees were assessed by morphological and behavioral characteristics (Boesch et al., 2000; Goodall, 1986; Van Lawick‐Goodall, 1968).

### 2.3 Coding

We developed an ethogram through reviewing the videos and with reference to prior descriptions of manual actions and grips in the literature. Videos were coded frame by frame. Our ethogram concerned (a) actions including insertions and subsequent extractions of the fishing probe, and while inserted, oscillations and positional adjustments of the probe, and other actions directed toward the probe or the termite nest, including forms of collecting and eating the termites from the probe (11 actions); (b) hand(s) used for each portion of the action sequence and whether they were used to grip or assist; (c) postures of the arms and hands; (d) height of nest opening with respect to eye level; (e) outcomes of attempted insertions; and lastly, (f) participation of the digits in grips and in assistances. Variables (a) through (e) are defined in Table 1. Participation of the digits in grips and assistances are defined below. Our goal in coding actions was to separate the continuous stream of activity with probes, termites, and the nest into discrete units for analysis. In our ethogram, an action is defined as the smallest functional unit of behavior that may appear in variable order with other actions.

**Table 1:** Variables and their definitions for the several categories of actions, modifiers of actions (hands used, posture of arms, position of hole with respect to the eyes), and outcomes of attempted insertions coded in this study

Variable	Definition
*Actions during fishing*	
Insert	Tip of probe penetrates the nest. Insert endures until the probe is extracted
Extract	Tip of probe exits the nest
Alternate	Hands alternate grip on the probe during insertion
Grip readjust	Release of grip during insertion followed by regripping with the same hand. Usually the second grip is more distal to the nest, resulting in a deeper insertion of the probe
Oscillate	Back and forth movement of the probe while it is inserted
*Actions removing termites from the probe*	
Direct eating	Chimpanzee eats termite(s) directly from probe after extraction
Sweep	Chimpanzee eats termite(s) gathered by sweeping the hand along length of stationary probe. After sweeping motion, termites are eaten from the sweeping hand
Slide	Chimpanzee eats termite(s) gathered by sliding probe through the assisting (stationary) hand. After sliding motion, termites are eaten from assisting hand. It is coded as a dry run if the sliding hand does not move to mouth after sliding
*Other actions*	
Straighten	Chimpanzee pulls probe through hands/mouth/fingers to straighten fibers
Switch	Changing the grip from one hand to the other while not inserting the probe
Open	Chimpanzee attempts to open termite tunnel exits on surface of nest with fingers
*Hand use*	
Unimanual	A single hand gripping the probe throughout the entire action
Bimanual	Concurrent or sequential use of both hands in relation to the probe (gripping or assisting)
Grip	Hand, foot or mouth holding the probe and actively controlling its movement
Assist	Hand touching the probe without gripping it
*Postures of arms and hands*	
Prone	Hand position where thumb points towards the body midline
Neutral	Halfway between prone and supine position; usually thumb points up
Supine	Hand position where thumb points away from the body midline
*Height of the nest opening with respect to eye level*	
At eye level	Probe angled at eye level
Above eye level	Probe angled above horizontal projection of the head
Below eye level	Probe angled below horizontal projection of the head
*Outcomes of attempted insertion*	
Failed insertion	Chimpanzee attempts to insert the probe, but it does not penetrate nest. Indicated by halted insertion movement
Successful insertion	Probe penetrates the nest

**Figure 1:**
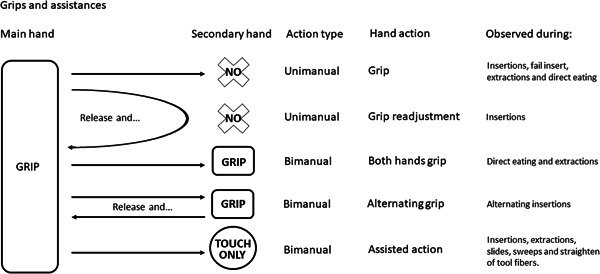
Grip and assistance diversity space during unimanual and bimanual actions. We did not observe any case where the foot was used to hold the probe

**Figure 2:**
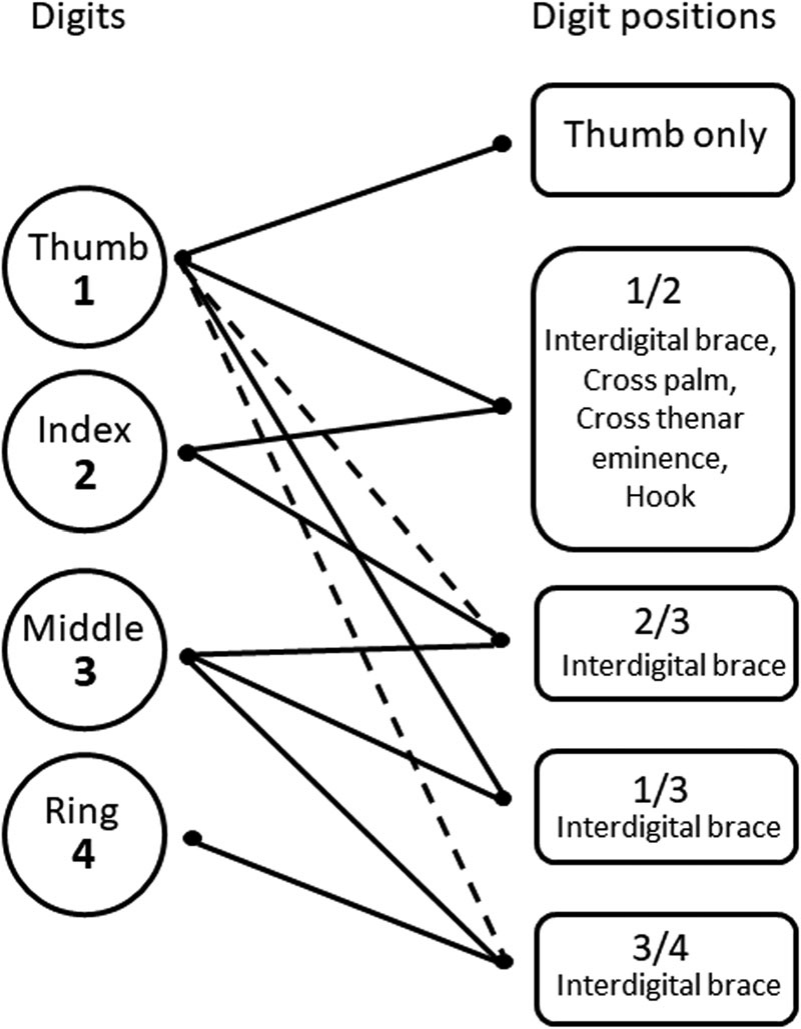
Diagram illustrating how the digit positions were coded. The dashed lines represent the optional use of the thumb (in most alternating insertions the thumb was used only for brief moments and on a single hand). There were no actions with the fifth digit in the sample. The phrases listed in each box are names provided by other authors for grips with that particular combination of digits (Lesnik et al.,^2015^; Napier,^1961^)

A general overview of the way grips and assists were coded is shown in Figures 1 and 2. Digit positions during grips and assists were indicated using numbers for each digit, with the thumb indicated as “1.” For example, 1/3 hand describes the grip of holding the tool between the thumb and middle finger. One video (of an adult, Maya) was excluded from the digit analysis due to low video quality. Bimanual grips were coded as symmetrical when the same digits 2–4 in both hands (2/3 or 3/4) contacted the probe and the position of the digits and movement of the hands were the same in both hands. Bimanual grips were coded as complementary when different digits 2–4 were used in the two hands. During symmetric bimanual alternating insertions, the hand closer to the body began and ended the action using the thumb (perhaps to stabilize the probe in the hand before changing the direction of its movement) whereas the thumb in the assisting hand was never used. The great majority of the action of bimanual alternating insertion, in time and space, occurred with neither thumb in contact with the probe. The quality of the video images constrained what we could code about manual actions; we could not, for example, reliably code which parts of the digits contacted the probe.

During the development of the ethogram, coders (A. O.‐M., M. O., E. C.) routinely discussed ambiguous cases to arrive at consensus. Following the development of the coding scheme, coders independently coded segments of video until interobserver agreements among all coders reached 85% agreement or better for actions and modifiers in line by line comparisons. Differences between coders in durations were calculated for each action sequence to the nearest 0.1 s and the differences summed for each video clip used for reliability calculations. Summed differences in duration were <1 s over each entire video clip (comprising multiple events with coded durations) in the final reliability corpus, indicating that observers coded onset and ending of events similarly. After the coding scheme was shown to be reliable, the video corpus was divided among the three coders for initial coding of actions and durations.

A. O.‐M. was designated the primary coder for final review of coding. A. O.‐M.'s intraobserver agreement for each variable, including digit positions, was between 96% and 100% on each of three videos used for this purpose. A. O.‐M. reviewed all the coding for all the video clips to confirm consistency.

The video corpus consisted of two to three video clips per individual in the sample (0:37 to 3:27 min; mean = 2 min). The videos were coded using Observer XT 10 (Noldus Corporation). We coded 1457 actions occurring before, during and after 440 attempted insertions. Every action included in the ethogram was observed several times in one or more individuals. Frequency count data were converted to rate per minute and tallied per individual. Distributions of actions within a category are presented as percentages. We report descriptive statistics for these variables. Durations during insertions with and without readjustments and with and without oscillations were compared using Wilcoxon matched pairs tests.

Two measures relating to hand use were derived from the data. First, handedness was calculated separately for unimanual actions and for complementary bimanual actions. For bimanual actions, the hand that initiated the movement of the probe was considered the primary hand for purposes of calculating handedness. We calculated a handedness index (HI) using the following formula: $\mathrm{HI}=\frac{r-l}{l+r}$ where “r” indicates the number of probe insertions with the right hand and “l” with the left. A positive value of HI indicates right‐handed bias, and a negative value a left‐handed bias, with possible scores ranging from +1.0 to −1.0. To measure the probability of an individual's degree of asymmetry, we calculated a *z* score for each individual as *Z* = $(r-0.5N)/\sqrt{0.25N}$, where *N* indicates the total number of insertions. Following the criteria discussed in Hopkins (2013), a “*z*” score above or below 1.96 (two‐tailed *p* < .05) indicated right or left handedness respectively. Second, Action Rate was calculated as the sum of all coded actions per minute. As each sliding action includes an extraction in our coding ethogram, we subtracted the frequency of slide from the sum of actions for calculation of the Action Rate, so as to count one action, not two, for sliding events.

## 3 RESULTS

We observed 440 attempted insertions, 378 (85.9%) of which succeeded in that the probe entered the nest. Of these, 338 (77.9% of successful insertions) were followed by eating termites, and 40 (10.6% of successful insertions) were not followed by feeding. On seven insertions we could see that the insertion was successful but could not see the hands clearly. Of the 371 insertions where the probe entered the nest and we could see the hands clearly enough, we observed 289 unimanual insertions (77.9%) and 82 bimanual insertions (22.1%; 78 with alternating grips and 4 with grip + assistance). We could classify 356 extractions as bimanual or unimanual. We observed 287 bimanual extractions (80.6%), and 69 unimanual extractions (19.4%). Thus, chimpanzees typically inserted the probe unimanually and extracted it bimanually. In three failed insertions, the probe entered the hole but it was damaged during the insertion. In the other 69 attempted insertions that failed, the probe contacted the nest, not the hole, resulting in bending or damaging the probe. Almost all of the 69 failed insertions (97.1%) were followed by a tool modification (straighten the brush fibers in 77.5% of cases). Inserting the probe was the only action in which the probe was occasionally damaged.

### 3.1 Insertions

The chimpanzees inserted the probe a median of 9.5 times/min (individual range 7.4–14.1; see Table 2). A probe remained inserted (from when the tip entered to when the tip reappeared) for median of 2.5 s (individual range 2.0–4.3 s). Individuals performed insertions in varied ways (see Table 3). All chimpanzees occasionally moved the probe in the nest using an oscillating movement before extracting it (2.6–32.6% of insertions). Every chimpanzee left the probe in the nest longer, on average, when she oscillated the probe compared to when she did not oscillate the probe (median of 4.0 vs. 2.0 s; *W* = 28, *n* = 7; *p* < .05, two‐tailed). For all individuals, durations of their insertions with grip readjustments were longer than for their insertions without readjustments (median = 4.0 and 2.3 s, respectively; *W* = 28, *n* = 7, *p* < .05, two‐tailed).

**Table 2:** Rate per minute of actions during termite fishing in five adult and two subadult female chimpanzees, and the cumulative rate per minute of all actions (“Activity”)

Individual	Preparation		Attempt		Feeding techniques		Other	Overall rate/min all activities
	Open tunnel	Straighten probe	Insert probe	Failed insert	Sweep termites	Slide termites	Switch hands	
Samantha	1.6	6.3	14.1	3	0	10.6	0.7	59.8
Sarah	2	7.9	11.8	3	0.8	6.7	2.9	53.7
Dinah	1.8	7.8	9.9	2	0	4.1	0.2	40
Theresa	0.3	7.3	8.7	2	1.3	2	0.7	40.3
Moja	2.1	2.9	7.4	0.7	2.9	1.9	0.5	28.1
Maya	0.2	4.8	9.5	0.6	1.9	1.4	0	33.4
Catherine	0.4	4.2	9.5	0.9	5.1	1	0.2	35.9
Median	1.6	6.3	9.5	2	1.9	2	0.6	40
IQR	1.7	3.6	3.1	2.3	2.95	5.3	1.6	20.3

**Table 3:** Actions during termite fishing: Percentage of all attempted insertions that succeeded mechanically (i.e., the probe penetrated the nest) for each individual, and for successful insertions, rate/min of insertions and percentage of insertions accompanied by oscillation, readjustment of the grip, and use of alternating hands for insertions

Individual	Succeed (%)	Insert/min	Oscillate (%)	Readjust (%)	Alternate (%)
Samantha	81	14.1	2.6	7.8	2.6
Sarah	79	11.8	5.7	37.1	12.9
Dinah	80	9.9	18.4	15.8	15.8
Theresa	81	8.7	3.8	7.7	7.7
Moja	91	7.4	29	6.4	9.7
Maya	94	9.5	32.6	15.2	6.5
Catherine	86	9.5	9.6	3.8	63.5
Median	81	9.5	9.6	15.2	12.9
IQR	11	3.1	25.2	25.2	9.3

Chimpanzees alternated hands gripping the probe (coded as insertion with alternating hands) on 2.6–63.5% of insertions. In some unimanual insertions (median, 7.8%; individual range, 3.8–35.7%), the grip of the hand was readjusted to a position on the probe more distal from the nest, to insert it deeper into the nest.

### 3.2 Extractions

**Figure 3:**
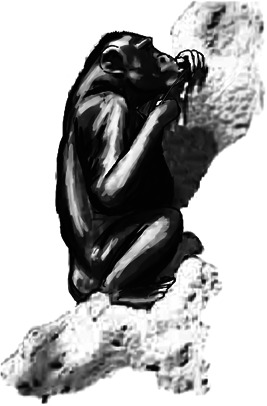
Sarah eating termites directly from the probe. This is the simplest method to retrieve termites from a probe. No concurrent manual actions are necessary

**Figure 4:**
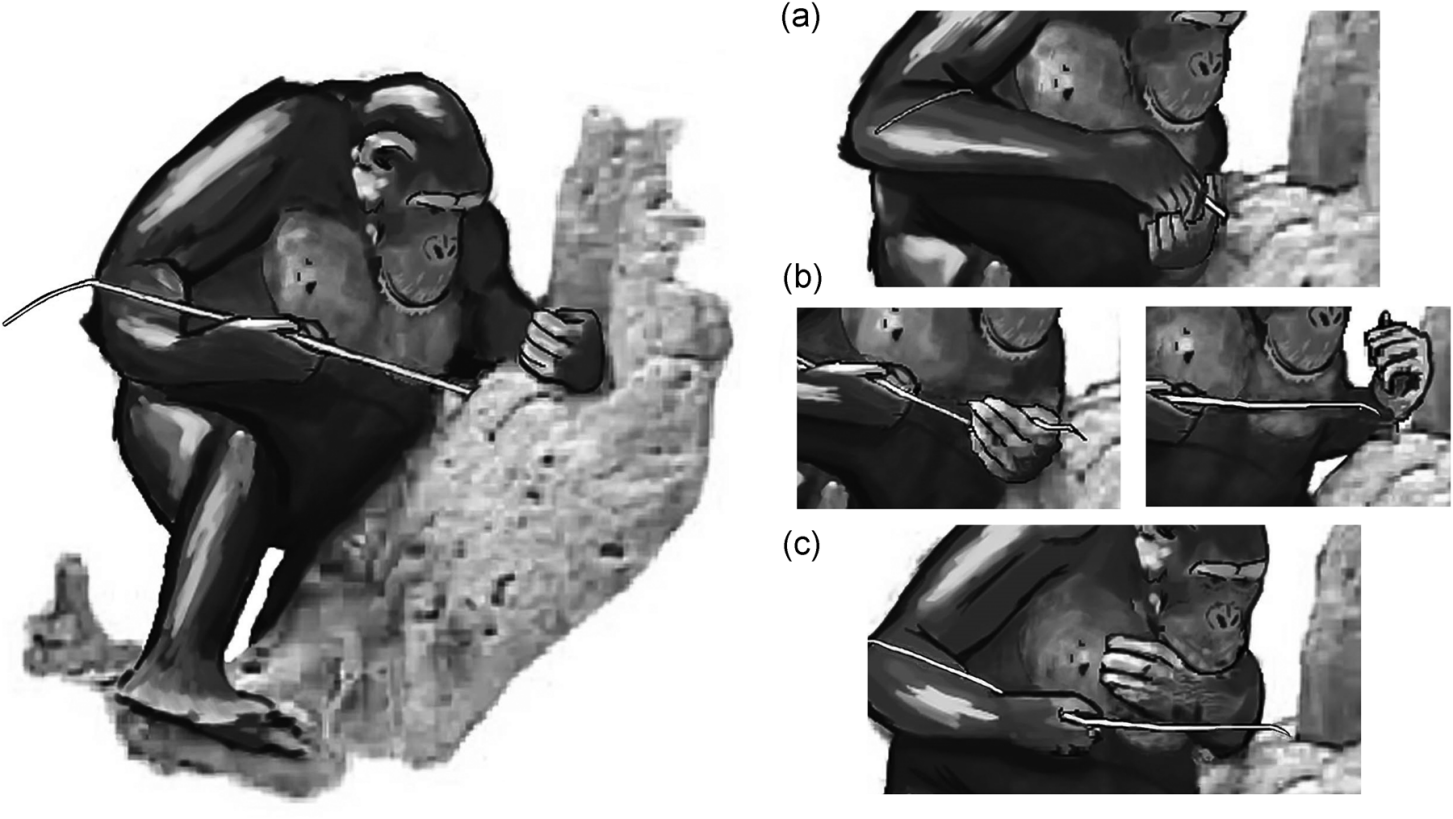
The sweeping technique. A bimanual coordinated action where (a) the probe is inserted with one or two hands, then (b, left) the probe is extracted, then (b, right) the assisting hand sweeps the probe and (c) termites can be eaten. This technique allows the chimpanzee to reinsert the probe while eating the termites

**Figure 5:**
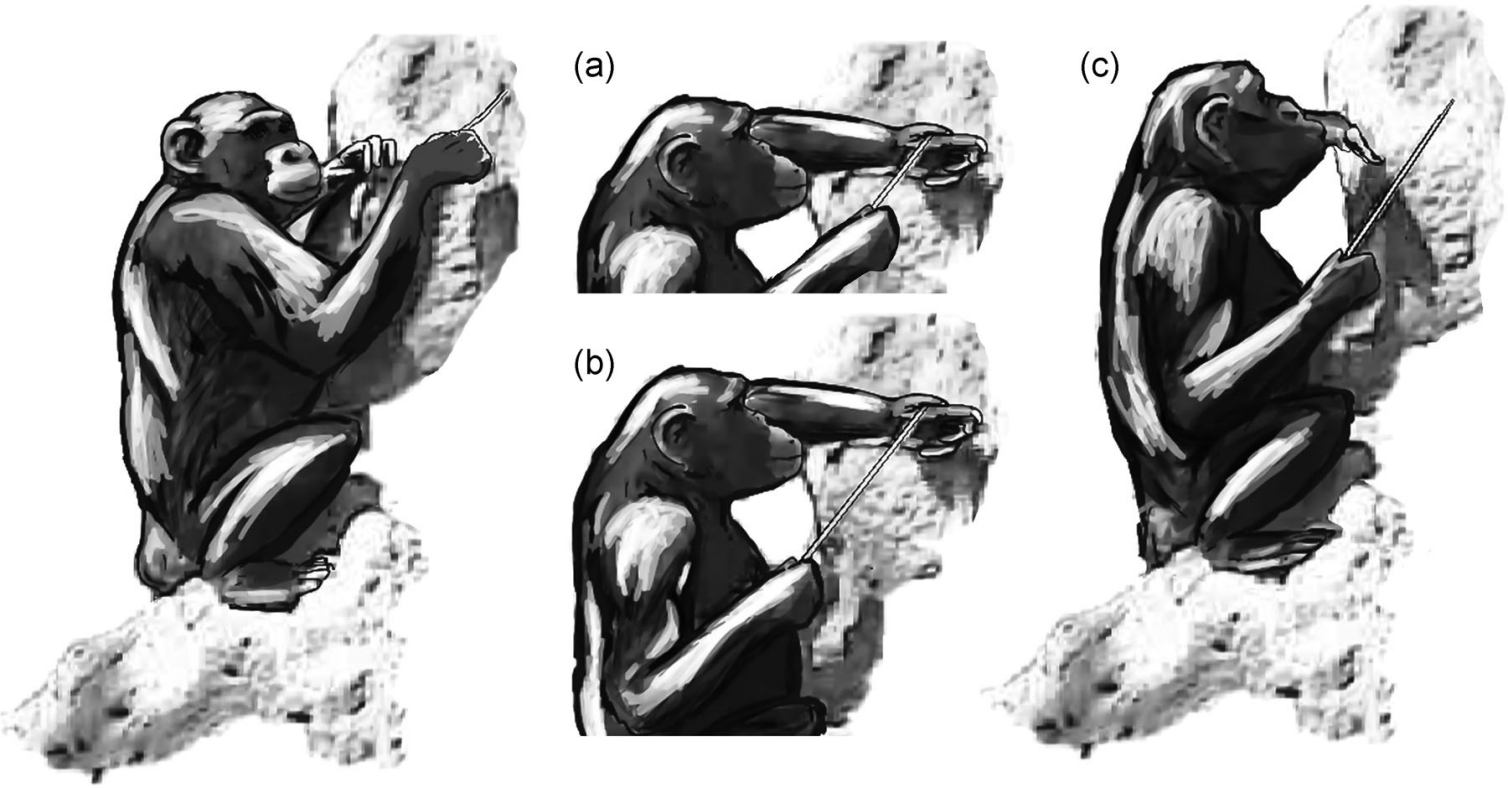
The sliding technique. A bimanual coordinated action usually done in an asymmetrical way, where (a) the probe is inserted, usually with an assisting hand close to the termite nest hole, then (b) extraction + sliding, always done in this way in our sample. One hand slides the tool between opposing surfaces of the assisting hand, usually between the thumb and the side of the palm, as the assisting hand lightly pinches the probe, and (c) termites are eaten from the assisting hand. This technique allows the chimpanzee to reinsert the probe while eating the termites

**Figure 6:**
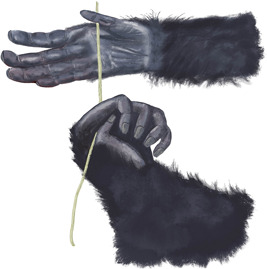
Thumb assist, as performed during the sliding technique. Sometimes the thumb's assistance changes to a grip, and both hands transport the probe to the mouth, resulting in eating termites directly from the probe

We observed three general ways of extracting and eating the termites:

1) Eating the termites directly from the end of the probe (called dip‐single and direct‐mouthing in ant predation; Humle & Matsuzawa, 2002; Sanz et al., 2010, 2014; Whiten et al., 2001; Yamakoshi & Myowa‐Yamakoshi, 2004): 42.1% of feeding actions. Only one hand is needed to perform the whole process. It appears to be a slow technique because it is a step by step sequence that excludes overlapping actions (Figure 3). Two individuals used this technique for a majority of their extractions (Theresa and Maya).
2) Sweep the termites from the probe (called ant‐dip wipe and pull‐through in ant predation; Humle & Matsuzawa, 2002; Sanz et al., 2010; Whiten et al., 2001; Yamakoshi & Myowa‐Yamakoshi, 2004): 17.5% of feeding actions. Sweep is a complementary bimanual movement that allows some overlapping actions. The individual can eat the termites at the same time that it is inserting the probe again (Figure 4). One individual used this technique for a majority of her extractions (Moja), and one at the same frequency as direct eating (Catherine).
3) Slide the termites from the probe: The slide is defined by moving the probe along a stationary assisting hand (40.4% of the probe feeding actions). The assisting hand is held close to the opening of the nest (Figure 5). The action was commonly done by moving the probe between the thumb and lateral side of the stationary palm (Figure 6). This thumb position was used almost exclusively (92.9%) in the sliding technique, and it occurred in a majority of sliding events (71% of thumb assistances during sliding technique). Only Sarah used other assisting positions during slide: she used fingers other than her thumb on 76.3% of her slides. Across individuals, only 4% of slides were not followed by eating (“dry run,” in Tables 1 and 4). The slide was the most frequently used technique by three individuals (an adult, Samantha, and the two subadults, Samantha and Dinah).

**Table 4:** Symmetric and complementary digit positions during bimanual actions

	Symmetric (*n*, %)	Complementary (*n*, %)	Total (*n*)
Insertions	16, 61.5	10, 38.5	26
Insert alternating	44, 83	9, 17	53
Extract	26, 27	70, 73	96
Direct	23, 31.5	50, 68.5	73
Sweep	10, 18.2	45, 81.8	55
Slide	23, 15.3	127, 84.7	150
Slide dry run	0, 0	6, 100	6
Straighten	54, 56.8	71, 43.2	125

### 3.3 Digit positions

**Figure 7:**
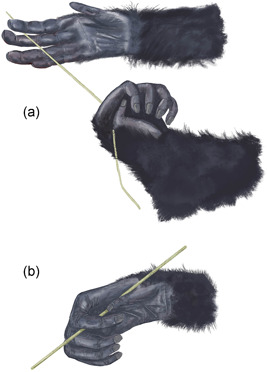
Comparison of a 2/3 NT (no thumb) grip/assist (right hand (a)), and the same hand position with thumb use (b)

A variety of digit positions were observed during insertions and extractions (see Table 5). During insertions, the most common digit position was 2/3, a grip without the thumb (60% of grips during insertions; 65% of grips during extractions; 48% of assists; see Figure 7a). This is partly due to the intensive use of the interdigital 2/3 by the chimpanzee with the highest proportion of alternating hand insertions (Catherine; 63.5% alternating insertions; see Table 3). This chimpanzee used digit position 2/3 for 98% of her alternating insertions. Although 3/4 was the second most frequently observed digit position used in the assisting hand during assists to bimanual insertions, this digit position was never seen in the gripping hand in bimanual insertions. It was used occasionally during unimanual insertions. Assisted bimanual insertions were not common (*n* = 13). Only four chimpanzees used assisted bimanual insertions but all seven females used some bimanual alternating insertions (*n* = 59). Due to low visibility, digit position during assisted bimanual insertions was coded only for three individuals. Interestingly, one of the two cases recorded of a 1/3 digit position occurred during an assisted bimanual insertion (the other one was used in a straightening action, by the same chimpanzee, Moja).

**Table 5:** Positions of the digits during grips and assistances during insertions and extractions, number of observations of the position and proportion among the sample, and number of individuals exhibiting these positions

A. Unimanual (289 unimanual insertions and 69 unimanual extractions)												
	Unimanual digit positions during insertions	
	1		1/2		1/3		2/3		3/4		Cr. Th.	
Grip n	/		60		/		146		38		/	
Grip %	/		24.6		/		59.8		15.6		/	
*N*_ind_ grip	/		3		/		6		2		/	
	Unimanual digit positions during extractions											
	1		1/2		1/3		2/3		3/4		Cr. Th.	
Grip n	/		7		/		44		4		/	
Grip %	**/**		12.7		/		80		7.3		/	
*N*_ind_ grip	**/**		2		**/**		4		1		**/**	
B. Bimanual (82 bimanual insertions and 287 bimanual extractions)												
	Bimanual digit positions during insertions	
		1		1/2		1/3		2/3		3/4		Cr. Th.
Grip n		0		6		1		13		0		0
Grip %		/		30		5		65		/		/
Assist n		0		6		0		12		7		0
Assist %		/		24		/		48		28		/
*N*_ind_ grip		0		1		1		3		2		0
*N*_ind_ assistance		0		1		0		3		1		0
	Bimanual digit positions during extractions	
		1		1/2		1/3		2/3		3/4		Cr. Th.
Grip n		0		64		0		151		40		1
Grip %		/		25		/		59		15.6		0.4
Assist n		137		25		0		4		88		0
Assist %		53.9		9.9		/		1.6		34.6		/
*N*_ind_ grip		0		4		0		6		2		1
*N*_ind_ assistance		4		5		0		3		5		0

*Note:* The number of cases per variable varies due to obscured events. Grip n: number of grips per type in the bimanual sample; Grip %: proportion of one position among the row; Assist: number of assistances with each digit position among the sample; Assist %: proportion of digit position among the row; N grip: number of individuals exhibiting the digit position during grips; N assistance: number of individuals exhibiting the digit position during assistances. Grips and assisting positions: (1) thumb only; (1/2) thumb and index; (1/3) thumb and middle finger; (2/3) middle and ring finger; (Cr. Th) cross thenar eminence.

During bimanual insertions, digit positions were generally symmetric, whereas during bimanual extractions, digit positions were primarily complementary (Table 4). Bimanual actions were mainly used during extractions (see Table 4.) During bimanual extractions, the interdigital position 2/3 was the most common grip (59%) and the single thumb assist 1 was the most common form of assistance (53.9%) (Table 5). This pattern reflects the frequent use of the sliding technique (usually performed by sliding the probe past the thumb during the extraction). Sometimes the thumb assist transformed into a grip during extractions. For example, an individual occasionally initiated a slide action with thumb assist but shifted the grip to the initially assisting hand and ate the termites directly from the probe; 1.8%), and a few times a chimpanzee used a single thumb grip during sweeps (2.9%) and as an assist while straightening the probe (2.4%).

The interdigital 3/4 position, a grip without the thumb, was the second most used during extraction grips and assistances. Two chimpanzees used this grip consistently. Catherine used 3/4 in most of her extractions, sweeps, straightening of the probe, and direct feeding actions. Sarah characteristically assisted the sliding technique with a 3/4 digit position, and used this grip during most of her insertions, feeding actions, and straightening actions.

### 3.4 Forms of bimanual action

Some bimanual actions could be done with one gripping hand and the other assisting, or with two gripping hands (simultaneous or dynamically alternating grips). Bimanually coordinated actions were performed with symmetrical and complementary (nonsymmetrical) digit positions. Bimanual insertions were typically symmetrical, especially among alternating insertions (61.5% of assisted insertions were symmetrical, and 83% of alternated insertions were symmetrical). Extractions and feeding actions were generally done in a complementary way (80% of extractions, 68.5% of feeding actions from the probe, 81.8% of sweeps, and 84.7% of slides). The most common correction of the probe, straightening the tool fibers, showed a more balanced proportion, with 56.8% symmetric events and 43.2% complementary events (see Table 6).

**Table 6:** Handedness: Percentage of insertions made with the right hand, total number of successful insertions (with right and left hands), HI and *z* values for total insertions

Individual	Samantha	Sarah	Dinah	Theresa	Moja	Maya	Catherine
Right hand	100	100	100	39	0	0	100
*n* (total)	115	70	38	26	31	46	52
HI	1	1	1	−0.23	−1	−1	1
*z*	10.64	8.37	6.16	−1.17	−5.57	−6.76	7.22
*p*	<.0001	<.0001	<.0001	.121	<.0001	<.0001	<.0001

Abbreviation: HI, handedness index.

On some occasions, grips and assistances were performed using the fingers without the thumb (coded as no thumb [NT] positions). In NT positions, typically the probe was supported between the medial and distal phalanxes of digits 3 and 4. NT digit positions were used as grips during 90.9% of insertions with alternating hands and seen in all individuals. Only one individual, Sarah, used NT digit assists during insertions (9.1% of her insertions).

### 3.5 Posture of the arm and hand

The most frequently used hand position during insertions was prone (54.7%), followed by neutral (41.9%), and supine (3.4%; see Table 4). As expected, hand positions during insertions varied as a function of the height of the targeted hole on the termite nest and the body position required to fish it (Table 7). Prone insertions were the most common at all eye levels. Neutral hand positions during insertions were common at eye level, and the least frequently used hand position, supine, was absent above eye level. Prone and neutral positions were coded more often during unimanual insertions than during bimanual insertions. Supine positions occurred more often during bimanual insertions than unimanual insertions (73.3% vs. 26.7%).

**Table 7:** Hand positions during insertion

Hand position	Prone	Neutral	Supine
Above eye level	9.3	3.4	0
At eye level	61.1	83.1	25
Below eye level	29.6	13.5	75
Sum	100	100	100
Overall	54.7	41.9	3.4
Bimanual	15.3	44	73.3
Unimanual	84.7	56	26.7
Sum	100	100	100

*Note:* Percentages of each hand position during insertions at different heights; overall percentage use of each hand position during insertions, collapsed across heights; and percentage of each position during unimanual and bimanual insertions at all heights.

The five adult females made more than half of their insertions at eye level, but the two subadults (Dinah and Samantha) made more than half of their insertions at other levels (see Table 8). Most successful insertions occurred at eye level (69.5%), with 6.7% above and 23.8% below eye level. The proportion of failed insertions at eye level was 13.5%, but below eye level 21.3% of insertions failed, and above eye level 30.3% of insertions failed.

**Table 8:** Number of observations and percentage of mechanically successful and failed insertions at different heights per individual

	Below		Eye level		Above		Overall success (%)
	Successful	Failed	Successful	Failed	Successful	Failed	
Theresa	‐	‐	26, 81.2	6, 18.8	‐	‐	81
Moja	3, 100	0, 0	28, 90.3	3, 9.7	‐	‐	91
Catherine	8, 80	2, 20	44, 93.6	3, 6.4	‐	‐	86
Maya	‐	‐	46, 93.9	3, 6.1	‐	‐	94
Dinah	11, 84.6	2, 15.4	13, 92.8	1, 7.2	14, 73.7	5, 26.3	80
Sarah	‐	‐	60, 82.2	13, 17.8	9, 64.3	5, 35.7	79
Samantha	58, 76.3	18, 26.7	53, 86.9	8, 13.1	‐	‐	81

*Note:* Each value is presented as “A, B”; where “A” refers to number of observations, and “B” to percentage.

### 3.6 Lateral bias

Six chimpanzees used one hand exclusively during insertions (4R, 2L). One (Theresa) used her left hand for 33% of insertions (see Table 6). All six chimpanzees that used one hand exclusively to insert the probe used exclusively the other hand to perform nontooling actions (e.g., manually opening a new hole while gripping the probe, mopping the surface of the nest while inserting the probe, or manually gathering termites while fishing). For the whole sample, most (72.5%) of the nontooling actions were done with the hand that was not preferred for inserting the probe.

### 3.7 Use of the mouth to assist

Chimpanzees did not grip the probe in the mouth during probing attempts, but they did use the mouth to assist in straightening the probe (4 times, 1 of them with obscured digit positions). In those cases, the individuals used one hand to grip and the other hand and mouth to assist. These actions might have been feeding attempts, or could have served straightening the probe and feeding concurrently.

### 3.8 Styles of termite fishing

Individual percentages of successful insertion ranged from 79 to 94 (see Table 6). Six of seven chimpanzees had a strong preference for using one of the two bimanual fishing techniques (slide or sweep). Three chimpanzees (Samantha, Sarah, and Dinah) used the slide more frequently than the sweep, and three others (Moja, Catherine, and Maya) used the sweep more frequently than the slide (see Table 2). Of the three chimpanzees that used slide more frequently than sweep, only Sarah used the sweep at all. All three sliders were right‐handed (Dinah, Sarah, and Samantha), as was one sweeper (Catherine). Two sweepers (Moya and Maya) were left‐handed. Theresa, the only chimpanzee without a strong bias for feeding technique, was also the only chimpanzee without a strong lateral bias, and one of two to feed directly from the probe proportionally more frequently than to use a bimanual method of collecting termites from the probe.

The fishing technique used by an individual seems to be related to other variables, in addition to lateral bias, resulting in what we suggest can be identified as different fishing styles (see Table 9). Compared to “sweeping” chimpanzees, “sliding” chimpanzees had a somewhat higher rate of successful insertions (sliders' rates: 13.9, 11.7, 9.9; sweepers' rates: 9.5, 9.5, 7.4), a higher rate of straightening of the probe (median, 7.8 vs. 4.2/min), and a higher rate of action overall (median, 51.2 vs 32.5 actions/min, respectively). Sliders performed more fishing actions below and above eye level, a higher percentage of readjustments during insertions (20.2% vs. 8.5%) and a lower percentage of oscillations (11% vs. 26%) than sweepers. Sliders are more versatile in where they insert the probe, but on the other hand, the rate of failed insertions for the three sliders was higher than for the three sweepers (3 vs. 0.7/min).

**Table 9:** Broad comparison of activities (expressed as rate per minute and percentage of insertions) for individuals classified as using one of two fishing styles

Styles	Sliders			/	Sweepers			
Individuals	Samantha	Sarah	Dinah	Theresa	Moja	Maya	Catherine	Mean
All actions/min	59.8	53.7	40	40.3	28.1	33.4	35.9	41.6
Insertions/min	14.1	11.8	9.9	8.7	7.4	9.5	9.5	10.1
Sweep/min	0	0.8	0	1.3	2.9	1.9	5.1	1.7
Slide/min	10.6	6.7	4.1	2	1.9	1.4	1	4
Direct eat/min	6.9	2.7	2.9	5.3	1.7	4.4	5.1	4.1
Failed insert/min	3.2	3	2	2	0.7	3	5	2.4
Straighten/min	6.3	7.9	7.8	7.3	2.9	4.8	4.2	5.9
Oscillate (%)	3.5	5.7	18.4	3.8	29	32.6	9.6	14.6
Right hand (%)	100	100	100	33	0	0	100	/
Above or below eye level (%)	48.7	14.3	65.8	0	9.7	0	35.3	24.8
Readjust (%)	7.8	37.1	15.8	7.7	6.4	15.2	3.8	13.4
Successful insertion (% of attempted)	81	79	80	81	91	94	86	84.6

*Note:* Theresa shares characteristics of both styles and is placed in the middle of the table.

Individual variations were also evident. Catherine inserted the probe with alternating hands proportionally more frequently than others (55.9% of all the alternating hands insertions belong to Catherine). While most sliding actions were done using the thumb for assistance (80.1% of sliding events; Figures 5 and 6), one chimpanzee used a 3/4 assistance when sliding the probe in 77.5% of her sliding events.

Individual variations in positions of the arm and digits were not evident. All chimpanzees used predominantly prone and neutral positions at eye level and above, but supine positions when fishing below eye level.

Three fishing sequences are presented with the supplementary material, all of them with a different insertion and feeding technique. Video S1 shows a sequence of insertion with grip readjustments and direct eating (also shown in Figure 3). Video S2 shows an insertion with alternating hands followed by a sweeping feeding action (also shown in Figure 4), and Video S3 shows a simple unimanual insertion with a sliding feeding action (also shown in Figure 5).

## 4 DISCUSSION

We describe the actions and postures that female chimpanzees use to collect termites (*M. muelleri*) from epigeal nests, attempting to address some of the dimensions of manual dexterity described for humans (the position of the digits while grasping, the repertoire of manual actions, interlimb coordination, positional, and postural features of the hands and arms). Fishing for termites in the chimpanzee manner requires delicate, precise actions with a flexible probe (a length of a plant stem or twig) inside an obscured, irregular tunnel (in the termite nest) to capture small mobile prey. Environmental constraints in this task include the properties of the plant probes, the characteristics of the termites, and the structure of the termite nest. Organismic constraints include the size, perceptual and motor characteristics, movement capacities, attention span, and other features of the individual chimpanzee. Task constraints include the necessity of inserting the probe precisely into a small opening in the nest, provoking the termites to attach to the probe, then withdrawing the probe with the termites still attached to it, and finally transferring the termites to the mouth. We are able to describe only a small portion of this complicated system (digital positions, positional and postural features, and to some extent multidigit synergies and interlimb coordination).

### 4.1 General character of termite fishing in Goualougo

Adult and subadult chimpanzees fishing for termites used a varied repertoire of highly practiced manual actions in diverse positions. All chimpanzees readjusted the grip on the probe during insertions and extractions, and inserted the probe with alternating hands. They achieved productive outcomes (eating termites) in nearly 90% of insertion‐extraction cycles with the probe, but they failed to insert the probe into an open tunnel on 15% of their attempts, usually damaging the probe in the process. Thus, termite fishing remains modestly challenging even for well‐practiced individuals. Some of the challenges arise from task constraints that we could not measure (such as the structure of the termite nest). However, we could look at where the chimpanzees attempted to insert the probe, and how they handled the probe. In accord with previous observations of adaptive grips used to perforate termite nests by chimpanzees in this population (Lesnik et al., 2015), we observed the female chimpanzees adjust grips, movements of the probe, and movements and positions of the body and hands during termite fishing. They used bimanual actions for some insertions but even more so for extractions, suggesting that extraction requires more effortful control than insertion. The hands were used in a complementary way in some bimanual actions and in a symmetric way in others. Chimpanzees occasionally repaired the tool, principally by straightening the fibers of the brush tip. These are all dimensions of dexterous action.

### 4.2 New elements of behavioral repertoire described in this study

Chimpanzees have been described using two general ways for removing insects from fishing probes and dipping wands to eat them (Humle & Matsuzawa, 2002; Sanz et al., 2014, 2016; Whiten et al., 1999, 2001; Yamakoshi & Myowa‐Yamakoshi, 2004): pulling the tool through the hand, sweeping the insects onto the hand, followed by eating them from the hand (referred as ant‐dip wipe, pull‐through, and sweep technique), and eating the insects directly from the end of the tool (referred as ant‐dip‐single and direct‐mouthing). When fishing for *M. muelleri*, Sanz et al. (2016) reported a “relatively equal prevalence” of eating the termites directly from the tool and by a sweeping method (Sanz et al., 2016). We distinguished two different techniques (sweeping and sliding) that likely have been previously lumped together as “sweeping.” Sliding is defined as using a stationary assisting hand (close to the hole in the nest) to slide the termites (usually with the thumb) from the tool onto the hand while the gripping hand moves the probe out of the nest. Six of the seven individuals in our sample used this method during the study.

In principle, the three methods of removing termites from the probe (eating them directly, sweeping after extraction, and sliding during extraction) afford different opportunities to perform concurrent actions. Eating termites directly from the tool allows no overlap of actions. Sweeping termites from the tool requires full removal of the tool from the nest, then removing the termites. The tool can be reinserted while eating termites from the assisting hand. Sliding allows the assisting hand to remove termites from the tool at the same time the tool is extracted; the termites can then be eaten while the tool is inserted again. Therefore, sliding termites from the tool saves one step in the sequence compared to sweeping, and could be quicker than sweeping. In turn, sweeping could be faster than eating from the probe directly. These different ways of collecting the termites were associated with different rates of feeding in our study, but not in the expected pattern. We found that sweeping was the slowest method (1.7/min); eating directly from the tool and sliding resulted in feeding at more than twice the rate of sweeping, and they were equivalent (4.1 and 4.0/min, respectively). Perhaps direct feeding happens most often when the termites bite in larger numbers—“when the fishing is good,” in other words. This is an aspect of termite fishing that warrants further investigation.

A second action that we add to the repertoire of termite fishing we termed Oscillation (see Table 1). All the individuals in our study oscillated the probe after it was inserted into the nest and before it was pulled backward, to extract it from the nest. For all the chimpanzees, oscillating insertions lasted nearly twice as long as insertions without oscillations. We could not see the termites on the probes, so we could not determine if chimpanzees collected a different number of termites per extraction with one technique versus the other, or if oscillation resulted in some other functional outcome. Perhaps oscillation provokes termites to defend their nest from the invading probe, leading to recruitment of soldier termites to attack the probe or to the attackers attaching more firmly to the probe.

### 4.3 Diverse use of digits in gripping

We observed chimpanzees using diverse grips and postures. The chimpanzees frequently used interdigital grips involving index and middle finger (2/3), as well as middle and ring finger (3/4), on some occasions without the thumb (especially during alternating insertions). The diversity of grips suggests careful control of the probe's movement, compared to the strong force but less precision used to perforate nests (Lesnik et al., 2015). The combination of thumb and index finger was not prominent in their handling of the probe. Most insertions with alternating hands use grips without the use of the thumb, and a high proportion of those grips were symmetrical (83%; e.g., main hand with 2/3, including momentarily use of the thumb, and secondary hand with 2/3 without the thumb). We did not observe 4/5 grips in our sample. It appears that the fifth digit plays a minor role in the grips used during termite fishing by the chimpanzees in Goualougo.

In comparison to grips described by Marzke et al. (2015), we saw rather different use of the thumb to press the probe against the palm. We did not see a “V‐pocket grip,” where the object is gripped in the flesh web between digits 1 and 2. Instead, the probe was supported along the palm as well as between digits 1 and 2. The other digits were not involved. Perhaps this could be called an “extended V pocket,” that occurs when a long, slender object is held in the web between digits 1 and 2.

Marzke et al. (2015) reported that chimpanzees in Mahale, when fishing for ants, on 90% of observed instances used a “2‐jaw chuck” grip, where the object is held between the thumb and index finger (side, pad, or dorsal edge). We did not see this grip in the Goualougo chimpanzees probing for termites. Perhaps the greater rigidity of the probes used in ant‐fishing by the chimpanzees in Mahale permits different grips than the flexible plant stems used by the chimpanzees in Goualougo, or perhaps finer control of the probe is needed to fish for termites than for ants.

Neufuss et al. (2019) provide a valuable comparative data set for gorillas (*Gorilla beringei beringei*) processing three kinds of plant foods (two defended with stinging hairs or other plant parts) in Bwindi Impenetrable National Park, Uganda. Some clear differences between the gorillas' actions and the chimpanzees' actions in Goualougo are evident. First, gorillas rarely shifted their grip on an object once they had grasped it, whereas this was common for chimpanzees inserting and extracting probes. Second, Neufuss et al. (2019) observed gorillas using a different assortment of grips than we saw among the chimpanzees handling plant probes, although some are very similar. For example, their “V‐pocket grip,” by its second meaning “Object held either in web between full thumb and side of flexed index finger or held only by the full thumb in web” seems similar to what we called thumb grip and thumb assist. However, their definition of V‐pocket includes the possibility of using the index finger, which they describe as flexed. In our observations of thumb assist and thumb grip, the chimpanzees' index finger is not involved, and it is relaxed, not flexed. However, in general, the resolution of the video images we coded did not permit as detailed an examination of digit positions as Neufuss et al. (2019) achieved. They noted as a general point that gorillas' hands are best adapted to forceful grasping, useful in both locomotion and manipulation. In contrast, the grips we observed during termite fishing by chimpanzees were not forceful. We conclude that we do not yet have a full accounting of the range of movements and grips achieved by apes. No doubt the size, shape, mass, texture, rigidity, and other properties of the objects that are gripped, and the goal of handling the object (e.g., to eat it, or to move it in relation to another object, such as a termite nest) strongly influence the nature of grips and hand movements. A full accounting of the movement and grip capabilities of the ape hand remains a goal for the future.

### 4.4 Use of an assisting hand

Actions of the nongripping hand are rarely described, but we found them to be a central feature of how chimpanzees fished for termites. Fishing for termites involves the introduction of a long probe into a narrow hole in the termite nest. The gripping hand usually holds the probe at some distance from the nest opening. Thus, a secondary hand is often used to support or guide the probe closer to the entrance to the nest. One might expect that an assisting hand would be used most commonly during insertions, but that was not the case. Nearly all insertions were performed without assistance, while nearly all extractions were completed with assistance (perhaps to support a probe with termites on it), and common feeding actions (sweep and slide) always involved the use of a second hand. Straightening the probe, another common action, required the use of two hands. The most common digit positions in an assisting hand were 2/3 and 3/4.

### 4.5 Interlimb coordination: Symmetrical and complementary actions

Chimpanzees typically used both hands in alternation to insert the probe, and when they did so, the digits were usually positioned in a symmetrical way in the two hands (excluding the thumb). Perhaps the symmetrical use of the digits of the two hands during insertions reduces the cognitive demands of rapid interlimb coordination, as suggested by Kelso et al. (1979) in a classic study of adult humans performing bimanual striking movements. Tang et al. (2015) showed that, for adult humans grasping or pointing at objects of variable size and distance, the sequential execution of grasps can borrow from movements of the same hand in the past and from the other hand, suggesting how symmetrical actions can be easier to perform.

In contrast, during extractions, the digits were usually used in complementary positions (as, e.g., in sliding, where one hand gripped the probe and the second hand served concurrently to guide the probe and collect termites). Interestingly, although straightening the fishing tool apparently required a similar movement as sweeping termites (without the subsequent eating action), when sweeping, chimpanzees kept the digits of the two hands in complementary positions most of the time, whereas to straighten the tool, chimpanzees used a relatively balanced proportion of complementary and symmetric digital positions. If our interpretation that symmetrical hand postures are more economical to produce is correct, then the balanced use of complementary and symmetrical positions in straightening, versus routine use of complementary positions during sweeping suggests that straightening the tool presents some additional challenges to performance than sweeping.

Unfortunately, it is beyond the scope of the current study to evaluate the consequences (in time taken to complete actions, or some other metric of efficiency) or the sequential pattern of chimpanzees' symmetric and complementary positions of the digits for different actions during termite fishing. One would need video images of higher resolution, supporting more detailed scoring, and a larger database, to attempt this task. Borel et al. (2017) supply a model for how this task might be accomplished. Borel et al. (2017) studied manual actions of humans making bamboo points using sharp flints at the level of detail that we still aspire to reach in studies of nonhuman primates in natural settings. Bardo et al. (2016) used the same detailed approach to study bonobos (*Pan paniscus*) using a stick to retrieve fruit puree through holes in a log, and using a stick to move a food item through a maze. The difficulty of viewing these actions and positions in detail is no doubt part of the reason that historically, analyses of nonhuman primates' manual actions during foraging have generally concerned functional outcomes, rather than the forms of movement (Fragaszy & Crast, 2016). Improving video technology and image analysis software can increase the possibilities for collecting the needed records.

### 4.6 Styles of fishing

As mentioned above, the three chimpanzees that used slide more than sweep had a higher rate of attempted insertions and higher overall rates of action. On the other hand, chimpanzees that used sweep more than slide had a lower rate of failed insertions, a higher percentage of mechanically successful insertions, higher rate of oscillating the probe, and lower rate of straightening the probe (commonly done after a failed insertion). Overall, sweepers could be characterized as relatively cautious, maximizing rates of success at a steady (slower) pace, and sliders as having a faster‐paced, more risk‐tolerant approach to fishing. Perhaps these styles of action will be evident in other foraging activities of these individuals, suggesting consistent individual differences in styles of engagement with the physical world, or perhaps they reflect differences in age or experience. In any case, our findings suggest that individual variability in techniques could, like lateral bias (Sanz et al., 2016), be associated with small but perhaps discernible differences in rates of successful insertion and feeding in termite fishing, and in other actions associated with this activity. Whether individual variations of this magnitude are biologically meaningful must be determined.

### 4.7 Environmental constraints: Termites, nests, probes

Environmental constraints in this task include the properties of the plant probes, the characteristics of the termites, and the structure of the termite nests, none of which we could measure in this study. We know that the species of termites to be collected affects how chimpanzees fish for them. The relatively short length of the mandibles of *M. muelleri* (the species eaten by the chimpanzees in this study) apparently permits them to be removed from the probe by an assisting sweeping/sliding hand without risk. Termites with longer mandibles, such as *Macrotermes lilljeborgi*, the other species of termites commonly eaten by chimpanzees at Goualougo, are typically eaten directly from the probe (Sanz et al., 2014). Within a given species of termite, the behavior of the termites likely also affects the chimpanzees' actions during fishing. In our study, chimpanzees often oscillated the probe while it was inserted in the nest. Perhaps the behavior of the termites (i.e., their tendency to attach to the probe) varies seasonally or with some environmental variable (e.g., ambient temperature, humidity) that we did not measure.

The characteristics of the nest impact the collection of probes to be used for fishing. According to Sanz et al. (2014), probes used to fish *M. muelleri* from epigeal nests are shorter (*M* = 44.1 cm) than those used to harvest *M. lilljeborgi* from subterranean nests (*M* = 51.1 cm) in Goualougo. Longer tools might require more frequent use of two hands than shorter probes (Boesch & Boesch, 1990; McGrew, 1974). If that is the case, a higher proportion of bimanual actions can be expected when chimpanzees fish for *M. lilljeborgi* or other termite species with subterranean nests compared to termites in epigeal nests. Future research could examine if a higher proportion of bimanual actions occur when chimpanzees fish from subterranean nests and/or use proportionally longer wands.

We do not know how the internal structure of the nest impacts fishing actions. For example, tunnels may vary in width and curviness, and the interior surfaces may vary in friability. No doubt all these features impact how a probe should be maneuvered through the tunnel.

Epigeal nests provide opportunities to fish across a large surface, often including a large vertical dimension. Chimpanzees can thus choose to fish using holes at different heights with respect to their eyes. As inserting the probe is usually visually guided, fishing above eye level is presumably more difficult than fishing at or below eye level. This appeared to be the case in our study. Some chimpanzees did fish above or (more often) below eye level, but for five of the seven individuals, far more than half of their insertions were at eye level. In addition, percentages of successful insertions were highest for all individuals at eye level, while percentages of successful insertions were lowest for insertions made above eye level.

In addition to the challenge of visually guiding the probe, inserting it above or below eye level required adjusting the posture of the arm and hand. Insertions above eye level were made predominantly with the hand in a prone position; below eye level, the predominant position was supine. At eye level, the predominant position was neutral. Probably all these dimensions of adjustments contribute to the relative ease or difficulty of fishing in a particular place using a probe with particular mechanical properties. The most general prediction we derive from consideration of this system is that fishing at or below eye level and with shorter probes will likely be easier than fishing above eye level or with longer probes.

Given the importance of chimpanzees for illuminating the phylogenetic origins of human characteristics, the widespread occurrence of termite fishing across populations of chimpanzees in different geographical regions (thus, facing varied constraints in this task), and the documented variations in the styles of fishing for termites across regions (Boesch et al., 2020), we should take up this challenge. For example, Musgrave et al. (2020) reported that young chimpanzees were more likely to request a probe from an adult at a termite nest, and to be given a probe, in Goualougo than in Gombe, Tanzania. They linked this difference in the behavior of adults towards the immature individuals to the relative simplicity of fishing in Gombe (with a single tool type) compared to Goualougo (with multiple tool types, which are made from select plant species and modified to be more efficient). They further linked the pattern of adults helping (“teaching”) immatures in Goualougo to the social transmission of behaviorally challenging traditions in chimpanzees. Our findings indicate several features of the fishing actions performed at Goualougo that could be considered as challenging for a novice (such as fishing above and below eye level, oscillating the probe, sliding the probe through the assisting hand, and straightening bent probes). Musgrave et al. (2020) depiction of the differences in the general complexity of termite fishing between Goualougo and Gombe leads to the prediction that the characteristic features of manual action during termite fishing in Goualougo identified in this study will be less prevalent or perhaps absent in Gombe. It will be most interesting to compare the patterns of manual actions used by chimpanzees during termite fishing in Goualougo with those used by other chimpanzee populations.

To conclude, we highlight the need for additional studies to refine our knowledge of chimpanzees' manual dexterity, and to address how varying environmental, organismic, and task constraints contribute to individual variation in manual actions in termite fishing. This is an important component of establishing the potential adaptive basis for tool‐assisted foraging skills in wild primates, and of establishing the role of social setting in the maintenance of local traditions. We hope that this report will encourage others to study the organism—task‐environment system of termite fishing, particularly environmental and task constraints that we were unable to address.

## CONFLICT OF INTERESTS

The authors declare that there are no conflict of interests.

## Supporting information

Supplementary information.

Supplementary information.

Supplementary information.

## Data Availability

The data that support the findings of this study are available from the corresponding author upon reasonable request.
